# Variations in the Origin and Branching Pattern of the Profunda Femoris Artery and Their Clinical Implications

**DOI:** 10.7759/cureus.106300

**Published:** 2026-04-01

**Authors:** Rajani Singh, Mamta Rani

**Affiliations:** 1 Anatomy, Uttar Pradesh University of Medical Sciences, Etawah, IND

**Keywords:** lateral circumflex artery, medial circumflex artery, obturator artery, profunda femoris artery, variation

## Abstract

The profunda femoris artery (PFA) is a branch of the femoral artery, and it gives off medial and lateral circumflex arteries. The PFA and its branches are of utmost use in preventing avascular necrosis of the femoral head, during catheterisation, and in reconstructive surgeries. Due to the immense clinical implications associated with this artery, the study assumes great importance. The aim of the study is to highlight the varied anatomy of the PFA and its branches, along with correlating them with clinical implications. The literature was explored using the databases PubMed, MEDLINE, Wiley Online Library, and Google Scholar. Various terms related to the artery were used for the literature search. The literature revealed that the PFA and its branches vary greatly in various populations. The varied anatomy of this artery is of great use to vascular surgeons, to anatomists, and to oncologists.

## Introduction and background

**Figure 1:**
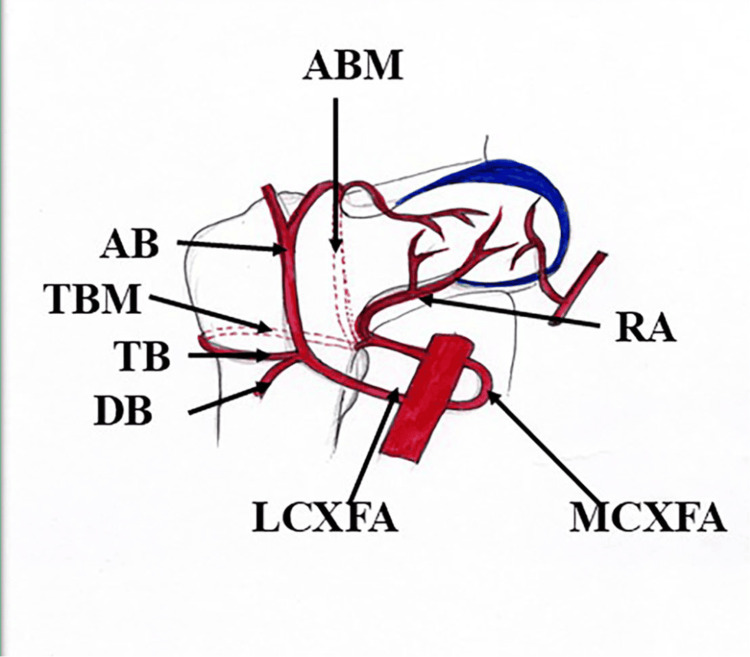
Normal origin of the PFA from the femoral artery and the origin of the medial and lateral circumflex femoral arteries and perforating arteries from the profunda femoris artery. LCXFA: lateral circumflex femoral artery; AB: ascending branch of the lateral circumflex femoral artery; TB: transverse branch of the lateral circumflex femoral artery; DB: descending branch of the lateral circumflex femoral artery; MCXFA: medial circumflex femoral artery; ABM: ascending branch of the medial circumflex femoral artery; TBM: transverse branch of the medial circumflex femoral artery; RA: retinacular arteries (branch of the medial circumflex femoral artery) Credit: The author, Dr. Rajani Singh.

The femoral artery is the main artery of the lower extremity, with three superficial and three deep branches placed in the femoral triangle. The profunda femoris artery (PFA) is the chief and largest deep branch of the femoral artery (Figure 1) originating on the lateral aspect about 3.5-4 cm distal to the inguinal ligament [1,2]. The PFA, also known as the deep femoral artery, irrigates all three compartments of the thigh, including adductors, extensors, and flexor muscles. The artery under study classically gives off medial circumflex femoral (MCXFA) and lateral circumflex femoral (LCXFA) arteries and three perforating arteries (Figure 1). The PFA continues as the fourth perforating artery [1,3]. The PFA not only varies in the site of origin, but also varies in the pattern of branching. The PFA, after its origin, curls medially and courses posterior to the femoral artery and vein, anterior to the pectineus muscle, then it passes through the space between the pectineus and adductor muscles, entering the adductor compartment. After this, the PFA passes downwards between the adductor longus and adductor brevis and then between the adductor longus and adductor magnus. Close to mid-thigh, the PFA continues as the fourth perforating artery by passing through the adductor magnus muscle [1].

**Figure 2:**
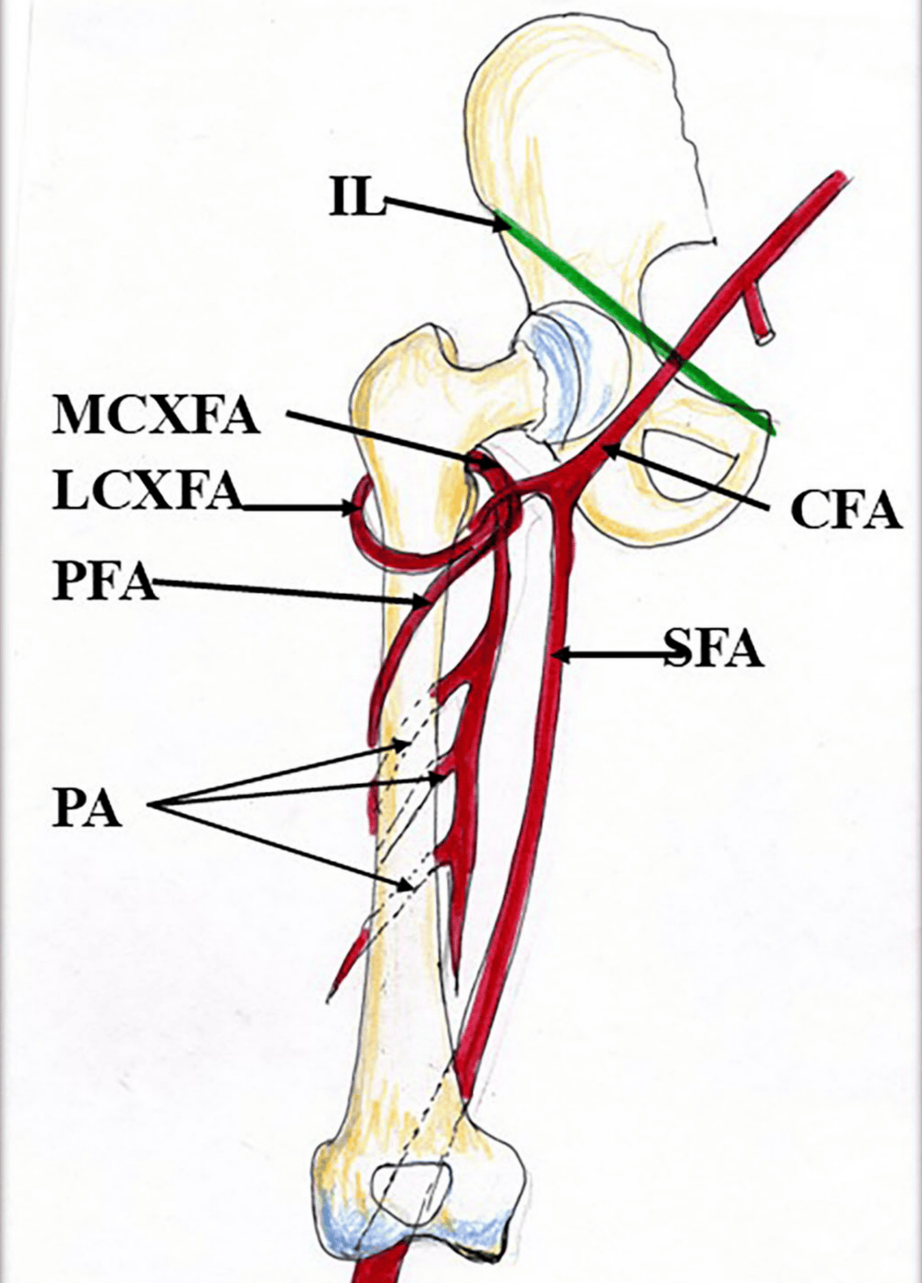
Main branches of the medial and lateral circumflex femoral arteries. IL: inguinal ligament; MCXFA: medial circumflex femoral artery; LCXFA: lateral circumflex femoral artery; PA: perforating arteries; PFA: profunda femoris artery; CFA: common femoral artery; SFA: superficial femoral artery Credit: The author, Dr. Rajani Singh.

The MCXFA surrounds the superior part of the femur, passing through the femoral triangle, adductor compartment, and gluteal region. Three branches, namely the transverse branch, ascending branch, and acetabular artery (Figure 2), sprout from the medial circumflex artery [1]. The transverse branch participates in the cruciate anastomosis, and the ascending branch in the trochanteric anastomosis. The retinacular arteries from the ascending branch irrigate the major part of the head and neck of the femur [4].

The LCXFA, another branch of the PFA, leaves the femoral triangle by coursing deep to the sartorius muscle and terminates by trifurcating into the ascending branch, descending branch, and transverse branch (Figure 2). The ascending branch anastomoses with the superior gluteal artery and deep circumflex iliac artery, forming the spinous anastomosis. The descending branch anastomoses with the lateral superior genicular branch of the popliteal artery around the knee joint. The transverse branch participates in the cruciate anastomosis [1]. Thus, circumflex arteries irrigate all three compartments of the thigh, including the upper end of the femur [4].

The superior three perforating arteries irrigate the hamstring muscles. These three perforating arteries anastomose with each other. In addition, the first perforating artery participates in cruciate anastomoses, the second perforating artery gives a nutrient artery to the femur, and the fourth perforator ends by anastomosing with the superior muscular branch of the popliteal artery [1].

The PFA forms the chief collateral route by anastomosing with the geniculate branches of the popliteal arteries and the recurrent tibial arteries. The knowledge of the site of the origin of the artery is essential in profundoplasty [5]. The PFA artery may be damaged by blunt trauma to the thigh in addition to fracture of the femur, including during surgical interventions involving fixation of metallic screws in the femur [6]. In all aforementioned clinical conditions, the PFA may be injured, leading to massive haemorrhage. Hence, a detailed comprehension of the distance of origin, source of origin, and branching pattern of the PFA is of utmost use to prevent pre- and post-operative complications. Hence, the study was taken up. This study aims to highlight and expound the normal and varied anatomy of the PFA and its branches, correlating with clinical implications.

## Review

**Figure 3:**
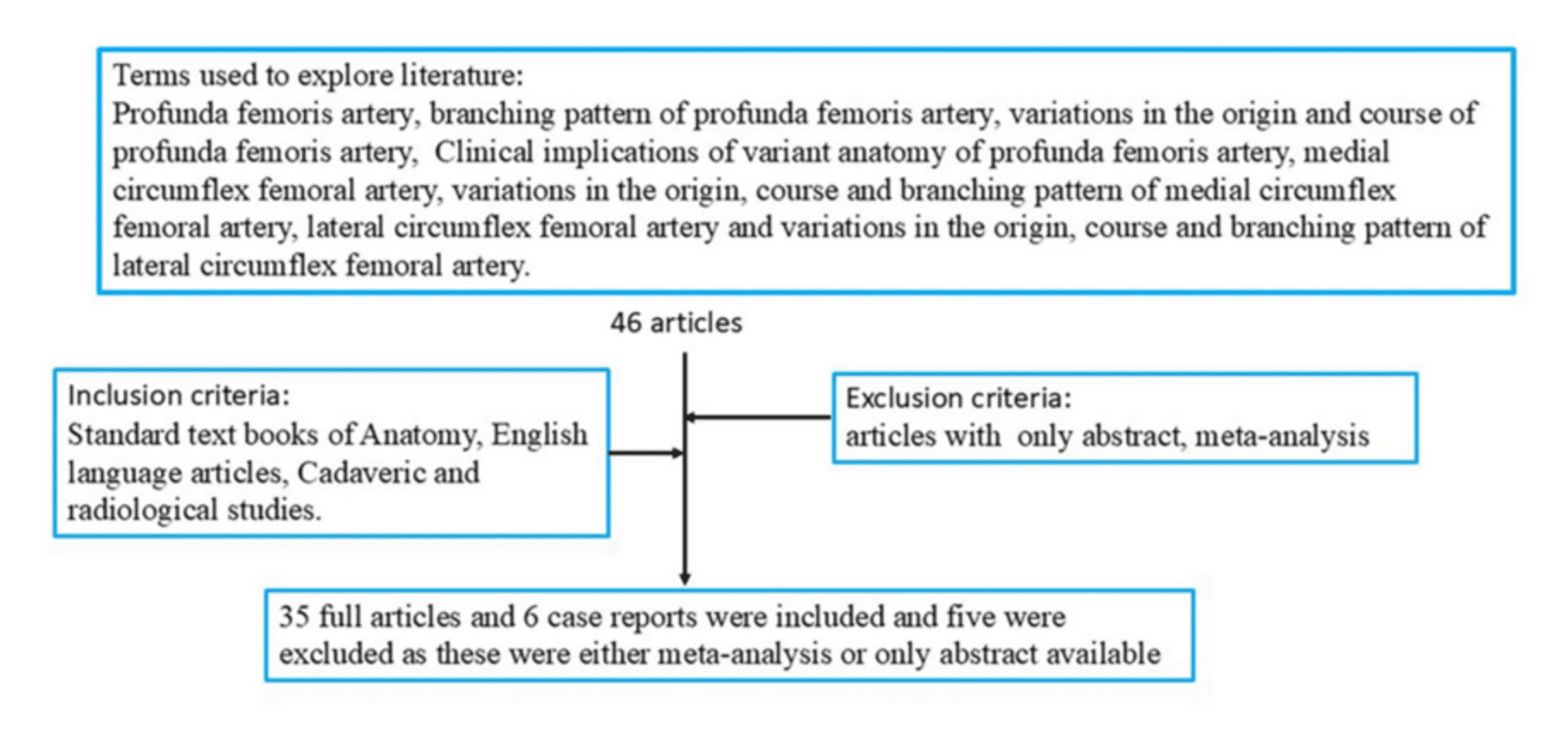
Flowchart summarising the search strategy. Credit: The author, Dr. Rajani Singh.

The study was conducted in the Department of Anatomy, Uttar Pradesh University of Medical Sciences, Etawah, India. The literature was surveyed using databases such as PubMed, SciELO, ResearchGate, Google Scholar, MEDLINE, Scopus, and Wiley Online Library. Standard textbooks of anatomy, including Gray’s Anatomy and Cunningham’s Manual of Practical Anatomy, were also examined. Only English-language articles were included. A flowchart summarising the search strategy is provided in Figure 3.

The data were collected, consolidated, and interpreted; pitfalls during various clinical procedures were recorded, and ways to correct them were elaborated in light of available literature.

Results and discussion

The PFA not only varies in the site of origin and course but also in the branching pattern. These variables related to the PFA are expounded in the following sections.

Variant Origin of the PFA

As per classical description, the PFA is described to originate from the lateral aspect of the femoral artery about 3.5-4 cm distal to the inguinal ligament [1,2]. But the origin of the PFA is found to originate from different aspects of the femoral artery. It is found to originate from the posterolateral aspect of the femoral artery in 50% of cases [5], in 53.03% of cases [7], and in 50% and 70% cases on the right and left sides, respectively [8].

The artery was observed to arise from the posterior aspect of the femoral artery in 20% of cases [9], in 46.9% of samples [5], and in 10.61% cases [7]. The vessel has been reported to arise from the posteromedial aspect of the femoral artery in 13.63% of samples [8], from the medial aspect in 3.03% of limbs [7] and 3.1% of cases [5], and from the anteromedial aspect in 1.51% of samples [7].

Distances of the PFA From the Mid-point of the Inguinal Ligament

Distances of origin of the PFA from the midpoint of the inguinal ligament are found to vary, as reported in different studies. These distances, as observed by different authors, are 3.2 cm [8], 3.75 cm [7], 4.2 cm [5], 5 cm [10], and 5.1 cm [9]. The site of origin of the PFA from the femoral artery is important, as it may lead to clinical conditions such as bleeding and arteriovenous fistula formation [11]. In these studies, the origin of the PFA is within the range described in classical anatomy. However, there is a case report in which the PFA arose very close to the inguinal ligament, with the distance of origin being 1.5 cm [12].

Normal and Variant Course of the PFA

Typically, the PFA curls medially after its origin and travels posterior to the femoral artery and vein, while being situated anterior to the pectineus muscle; subsequently, it traverses the area between the pectineus and adductor muscles, entering the adductor compartment [1]. Following this, the PFA descends between the adductor longus and adductor brevis, and then between the adductor longus and adductor magnus [1]. Near the mid-thigh region, the PFA continues as the fourth perforating artery by passing through the adductor magnus muscle [1]. The PFA is found to follow this standard course with almost no variation in its course, which is found in the literature.

The information regarding the varied anatomy, including the site of origin of the PFA, is essential in preventing iatrogenic femoral arteriovenous fistula while performing femoral artery puncture, as the PFA is to be identified while carrying out an incision for exposing the femoral artery and PFA [12]. Sahin et al. recommended high-resolution ultrasonic imaging prior to the catheterisation of femoral vessels and surgery in the femoral triangle to get detailed information about PFA and femoral vessels [13]. In addition, knowledge of higher origination of PFA is essential during interventions like femoral arterial and venous puncture and femoral nerve blocks, as these anatomic structures are closely related to each other in the femoral triangle [14]. There is a possibility of pseudoaneurysm formation if the exact site of origin of PFA is not known and PFA or the femoral artery is to be punctured [12].

The PFA anastomoses with the geniculate branches of the popliteal artery and the recurrent tibial arteries, leading to collateral circulation [5]. This information is important in surgical interventions involving profundoplasty [5]. In addition, this artery is closely related to the femoral shaft and may be injured during fixation of metallic screws in the femur [6]. Moreover, the PFA is the main vessel of the thigh used in arteriography, ultrasound, and Doppler imaging, digital subtraction angiography, and magnetic resonance imaging (MRI) [8]. Knowledge of the direction of origin of the PFA is also used in introducing catheters, in preparing pedicled flaps, and during reconstructive surgery [8]. Currently, the PFA is also used in haemodialysis in place of the superficial femoral artery.

Variation in the Branching Pattern of the PFA

The PFA classically gives off the MCXFA and LCXFA and three perforating arteries, and it continues as the fourth perforating artery.

Varied Anatomy of the Medial Circumflex Artery

The MCXFA is known by different names, such as the circumflexa femoris interna or arteriae circumflexae femoris medialis [1]. The MCXFA arises from the posteromedial aspect of the PFA in the femoral triangle [1]. However, this artery is subject to many variations, including origin from the common femoral artery, the common femoral artery as a common trunk with the PFA, a common trunk with the external iliac artery, and the common femoral artery as a common trunk with the LCXFA and superficial femoral artery [8]. The most common site of origin of the MCXFA is from the PFA, as described in standard textbooks of anatomy. It has been reported to originate from the common femoral artery in 40% and 35% of cases on the right and left sides, respectively [8], in 33.3% of cases [15], in 31% of samples [16], in 11.7% of cases [17], and in 32% of cases [5]. Some reports describe the origin of the MCXFA as a common trunk with the PFA in 15% of cases [8], in 2.4% of samples [15], and in 1.6% of cases [17]. There is also a study in which the MCXFA was found to originate from the common femoral artery as a common trunk with the LCXFA in 1.6% of cases [17]. Reports have also described the origin of the MCXFA from the superficial femoral artery in 3% and 5% of samples [16,17], and from the external iliac artery [18].

Another variation is the origin of the MCXFA and LCXFA as a common trunk from the PFA, observed in 23% of cases [19] and in 6.8% and 7% of samples [20,21]. There is a case in which the MCXFA arose from the femoral artery as a common trunk with the inferior epigastric artery and obturator artery [21]. The origin of the LCXFA from the PFA proximal to the origin of the MCXFA has also been observed [9].

In about 2.8% of specimens, the MCXFA was found to be absent [22,23]. The MCXFA has been reported to arise from the posterolateral aspect of the PFA [24], in contrast to the normal posteromedial aspect. In another study, the MCXFA was observed to arise from the lateral aspect instead of the normal posteromedial aspect [25].

As the course of the MCXFA is concerned, very few studies describe its variant course. During its variant course, the MCXFA may pass anterior to the femoral vein instead of posterior [26], and it may course lateral to the femoral artery instead of medial to it, or may pass between the pectineus and adductor longus [25].

Branching Pattern of the MCXFA

Normally, the MCXFA gives off three branches, namely the transverse branch, ascending branch, and acetabular artery [1]. The transverse branch anastomoses with the first perforating artery, the inferior gluteal artery, and the transverse branch of the LCXFA, forming the cruciate anastomosis [1]. The ascending branch of the MCXFA forms the trochanteric anastomosis and a vascular ring around the neck of the femur. Retinacular branches from this vascular ring supply the head and neck of the femur, and the acetabular artery supplies the femoral head [1]. Literature describing the variant branching pattern of the MCXFA is lacking.

Significance of the Varied Anatomy of the MCXFA

Since the MCXFA is the chief supply to the head and neck of the femur, knowledge of its varied anatomy helps in limiting avascular necrosis of the femoral head during embolisation procedures [27]. In addition, this information is useful to radiologists and orthopaedic surgeons during total hip arthroplasty [27], and to vascular and plastic surgeons when preparing vascular pedicles such as the transverse upper gracilis flap, the superomedial thigh flap, and the medial circumflex femoral perforator free flap, to reduce iatrogenic injury to the artery [20,28]. A varied course of the MCXFA may lead to an iatrogenic arteriovenous fistula during endovascular interventions such as cardiac catheterisation [26]. Orthopaedic surgery in the thigh involves a femoral nerve block, in which the anaesthetic solution is administered near the inguinal crease lateral to the femoral artery [29]. In such cases, an artery originating from the femoral artery and lying lateral to it may be iatrogenically injured, causing intraoperative bleeding and postoperative complications [29,30]. The incidence of such injury is reported to be 10-15%, which is relatively high [29], warranting surgeons to be aware of the varied anatomy of the MCXFA. The varied anatomy of the MCXFA is important in preventing femoral head avascular necrosis during surgical interventions for hip and acetabular fracture fixation by the posterior approach, as well as in performing both trochanteric and intertrochanteric osteotomies [30]. Variations in the anatomy of the MCXFA may be attributed to differences in samples from various races and populations with varying genetic factors [20].

The MCXFA and LCXFA form a substantial anastomosis around the hip joint, creating another route aiding surgeons to use these arteries for replacement surgeries involving the coronary artery, branches of the aorta, and popliteal artery [31].

Variant Origin, Course, and Branching Pattern of the LCXFA

**Figure 4:**
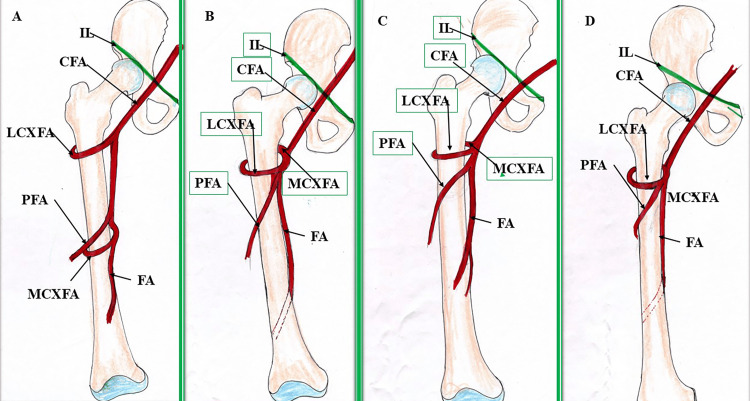
Variant origins of the LCXFA. (A) LCXFA arising from the common femoral artery; (B) LCXFA arising as a common trunk with the PFA; (C) LCXFA arising as a common trunk with the MCXFA; (D) LCXFA trifurcating with the MCXFA and PFA. IL: inguinal ligament; CFA: common femoral artery; LCXFA: lateral circumflex femoral artery; MCXFA: medial circumflex femoral artery; PFA: profunda femoris artery; FA: superficial femoral artery Credit: The author, Dr. Rajani Singh.

The LCXFA is another branch of the PFA, originating from its lateral aspect. However, it may arise from the common femoral artery, the common femoral artery as a common trunk with the PFA, the common femoral artery as a common trunk with the MCXFA, as a trifurcation with the MCXFA and PFA, or from the superficial femoral artery (Figure 4) [8,17]. Most commonly, the LCXFA arises from the PFA, with varying incidences [5,8,17,21], followed by the common femoral artery [5,8,17]. The second most common site of origin of the LCXFA in one study was from the common femoral artery as a common trunk with the PFA [21]. The LCXFA has been observed to arise from the common femoral artery as a common trunk with the PFA, with incidences of 5% and 1.6% in two studies [8,17], which are lower than that reported by Sinkeet et al. (10.7%) [21]. The LCXFA was also detected to originate from the common femoral artery as a common trunk with the MCXFA in two studies [17,21], with incidences of 1.6% and 14.3%, while the same artery was found to arise as a trifurcation with the MCXFA and PFA in one study [21], with an incidence of 7%. The LCXFA was also reported to arise from the superficial femoral artery in one study [9]. The LCXFA was observed to be absent in 3.3% of cases [17]. A duplicated LCXFA was observed by various authors [16,32,33].

Normally, the LCXFA gives off ascending, descending, and transverse branches (Figure 2) [1]. However, the literature shows that these branches may arise from the common femoral artery, as elaborated in the succeeding paragraphs.

The descending branch of the LCXFA was found to originate from the common femoral artery in 3.08% of samples [34]. In addition, at the trifurcation of the LCXFA, the transverse branch arose from either the ascending or descending branches [35]. Furthermore, two ascending branches were observed, one from the common femoral artery and the other from the PFA [35]. In one study, the ascending branch of the LCXFA was found to arise from the femoral artery [3].

Clinical Implications of the Varied Anatomy of the Lateral Circumflex Artery

Good knowledge of the LCXFA is useful during anterolateral thigh flap and tensor fascia lata myocutaneous flap reconstruction surgeries, as well as in robotic and laparoscopic surgery, to prevent arterial injury and flap necrosis [5]. The LCXFA and its branches may also be used in aortopopliteal bypass and coronary artery bypass grafting [36]. The ascending and descending branches of the artery are well used for vascularised iliac transplantation [37] and as an additional artery in extra-intracranial bypass surgery [21]. The varied anatomy of this artery is useful in hip joint replacement surgery and hip arthroplasty [19]. The variant origin of the LCXFA is important in administering anaesthesia to the femoral nerve. An anomalous origin of the artery is liable to iatrogenic damage during vascular procedures such as catheterisation, stenting, embolectomy, angiography, and angioplasty [38], as well as during posterior inferior cerebellar artery revascularisation and oropharyngeal reconstructions [39]. The variant origin of the LCXFA may alter the normal course of the artery and its topographic relationship with the femoral nerve in the thigh, causing failure of the femoral nerve block [33]. In cases of duplication of the LCXFA, the chances of nerve injury increase while obtaining the arterial pedicle due to the intimate relationship between the ascending branch of the LCXFA and the femoral nerve [32]. In addition, the LCXFA is used in facial reconstruction following gunshot wounds [40]. Recently, there has been increased use of these branches of the PFA as vascular pedicles during breast reconstruction after mastectomy in cases of carcinoma of the breast.

## Conclusions

The literature shows that the origin, course, and branching pattern of PFA and its branches vary substantially, as does ethnicity. The information on the origin, course, and branching pattern will be useful in preventing iatrogenic femoral arteriovenous fistulas during surgical treatments such as catheterisation, flap preparation with pedicles, reconstructive surgery, profundaplasty, and the fixation of metallic screws in the femur. The diverse morphology of MCXA is critical during total hip arthroplasty to avoid avascular femoral head necrosis, femoral nerve block, and acetabular fractures. Similarly, the variable origin, course, and branching pattern of LCXFA will be critical during antero-lateral thigh flap and tensor-fascialata-myocutaneous flap repair procedures, as well as robotic and laparoscopic surgery, to avoid artery injury.

Thus, precise and detailed knowledge of PFA and its branches will be useful to vascular surgeons carrying out diagnostic and therapeutic procedures around the femoral triangle, to radiologists for avoiding misinterpretations of radiographs, and to anatomists for rare variations relating to the anterior compartment of the thigh and to oncologists.
